# Assessment of Antifungal Efficacy and Release Behavior of Fungicide-Loaded Chitosan-Carrageenan Nanoparticles against Phytopathogenic Fungi

**DOI:** 10.3390/polym14010041

**Published:** 2021-12-23

**Authors:** Ravinder Kumar, Agnieszka Najda, Joginder Singh Duhan, Balvinder Kumar, Prince Chawla, Joanna Klepacka, Seweryn Malawski, Pardeep Kumar Sadh, Anil Kumar Poonia

**Affiliations:** 1 Department of Biotechnology, Chaudhary Devi Lal University, Sirsa 125055, Haryana, India; rsulakh@gmail.com (R.K.); pardeep.sadh@gmail.com (P.K.S.); 2 Department of Vegetable and Herbal Crops, University of Life Science in Lublin, 50A Doświadczalna Street, 20-280 Lublin, Poland; 3 ICAR-National Research Centre on Equines, Sirsa Road, Hisar 125001, Haryana, India; bmanuja.nrce@gmail.com; 4 Department of Food Technology and Nutrition, School of Agriculture, Lovely Professional University, Phagwara 144411, Punjab, India; 5 Department of Commodity Science and Food Analysis, Faculty of Food Science, University of Warmia and Mazury in Olsztyn, 2 Oczapowskiego Street, 10-719 Olsztyn, Poland; klepak@uwm.edu.pl; 6 Department of Landscape Architecture, University of Life Science in Lublin, 28 Głęboka Street, 20-400 Lublin, Poland; seweryn.malawski@up.lublin.pl; 7 Department of Molecular Biology, Biotechnology & Bioinformatics, CCS HAU, Hisar 125004, Haryana, India; anil_poonia2005@yahoo.com

**Keywords:** antifungal, nanocomposite, chitosan, bioactivity, polysaccharide, binary blend

## Abstract

Biopolymeric Chitosan-Carrageenan nanocomposites 66.6–231.82 nm in size containing the chemical fungicide mancozeb (nano CSCRG-M) were synthesized following a green chemistry approach. The physicochemical study of nanoparticles (NPs) was accomplished using a particle size analyzer, SEM and FTIR. TEM exhibited clover leaf-shaped nanoparticles (248.23 nm) with mancozeb on the inside and entrapped outside. Differential scanning calorimetry and TGA thermogravimetry exhibited the thermal behaviour of the nanoform. Nano CSCRG-1.5 at 1.5 ppm exhibited 83.1% inhibition against *Alternaria solani* in an in vitro study and performed as well as mancozeb (84.6%). Complete inhibition was exhibited in *Sclerotinia sclerotiorum* at 1.0 and 1.5 ppm with the nanoformulation. The in vivo disease control efficacy of mancozeb-loaded nanoparticles against *A. solani* in pathogenized plants was found to be relatively higher (79.4 ± 1.7) than that of commercial fungicide (76 ± 1.1%) in pot conditions. Nanomancozeb showed superior efficacy for plant growth parameters, such as germination percentage, root–shoot ratio and dry biomass. The nanoformulation showed higher cell viability compared to mancozeb in Vero cell cultures at 0.25 and 0.50 mg/mL in the resazurin assay. CSCRG-0.5 showed slow-release behavior up to 10 h. Thus, these green nano-based approaches may help combat soil and water pollution caused by harmful chemical pesticides.

## 1. Introduction

Alternative strategies and methods are in demand for the management of plant diseases and to minimize dependency on chemical pesticides and fertilizers. Fungi have devastating effects on plant growth and development due to their ability to grow in humid and varying environments and they reduce crop yields considerably. Approximately 30% of crop loss is due to insect pests [1]. The total loss, across the globe, due to pests is from 50% in wheat to over and above 80% in cotton. Other crops which suffer significant production losses due to pests are soybean (26–29%) as well as maize, rice and potatoes (31, 37 and 40%, respectively) [2]. 

*Alternaria solani*, *A. alternata*, *Stemplyleum lycopersici* and *Sclerotinia sclerotiorum* are destructive fungal pathogens that cause early blight, stem rot and leaf spot diseases in many vegetable crops, mainly tomato and potato. Chemical fungicides have been used and are the most potent means to control these fungal pathogenic diseases. However, regular use of chemical fungicides can be potentially harmful to animals and humans if their residue remains in the soil or the crops [3]. There are also drawbacks in terms of soil and water pollution [4]. Moreover, it has been found that the plants absorb only 0.1% of the applied chemical pesticides in field conditions. The remaining 99.9% is lost to the environment, causing adverse, detrimental effects in non-target organisms and polluting the ecosystem [5]. In recent years, nanotechnological interventions to combat these plant pathogenic diseases have gained attention due to their small size and targeted delivery for sustainable production, crop protection and disease management [6,7,8]. Diverse materials have been used alone or along with chemical fungicides, such as metal NPs [9], clays [10], hydrogels [11,12], polymers-metal conjugates [13,14,15] and biopolymers [16,17,18,19,20], to improve effectiveness against disease and protect the chemical pesticides from heat, moisture and premature degradation [21]. Chitosan nanoparticles have been used extensively among these polymers due to their biocompatible, biodegradable and non-toxic nature. 

Chitosan, a linear polysaccharide, is extracted from chitin, the main structural component of crustaceans (shrimp and crab) and some fungal cell walls. It maintains the immune system of plants, secretes enzymes and thereby improves disease and insect resistance. Similarly, carrageenan belongs to high-molecular weight sulfated polysaccharides. It is obtained from seaweeds and is commonly used as a thickening and emulsifying agent to improve the texture of commercial food products [22]. Carrageenan is a sulphated linear polysaccharide of d-galactose and 3,6-anhydro-d-galactose obtained by extracting certain red seaweeds from the Rhodophyceae class. The backbone of carrageenan has 3-linked β-d-galactopyranose units with 4-linked α-d-galactopyranose (d-units) or 4-linked 3,6-anhydro-α-d-galactopyranose (d-units). 

Mancozeb, propineb and thiram are among the top-selling fungicides, e.g., mancozeb sales are expected to reach $18 billion by 2025 [23]. Mancozeb (manganese ethylenebis (dithiocarbamate) polymeric complex with zinc salt), is a multisite activity fungicide that affects metabolism in target cells and can also affect bacteria involved in both C and N cycling in soil [24,25]. The European Commission established maximum residue limits (MRL) of 0.01–25 ppm for dithiocarbamates in various plants and products of vegetable or animal origin [26]. Excessive use of mancozeb has continued in recent years, e.g., amounts higher than the maximum residue limits have been detected in tomatoes [27], kiwi and pears [28]. Li et al. [29] reported 0.6–1.6 µg/kg and 0.8–2.5 µg/kg for mancozeb and propineb, respectively, in different vegetable food matrices. The present work was conducted to investigate the hypothesis that biodegradable and eco-friendly Chitosan-Carrageenan polymeric nanocomposite formation occurs due to the bond formed between chitosan’s positively charged amino groups and the anionic sulphate (SO_3_^−^) and hydroxyl group of carrageenan, with charges neutralized by sodium tripolyphosphate. This process gives rise to a self-assembled mancozeb-loaded polyelectrolyte nanomatrix of small size with a high surface area-to-volume ratio for site-directed sustained release, reducing environmental pollution and non-target toxicity compared to parent formulation.

According to the available literature, there is a lack of knowledge about Chitosan-Carrageenan nanocomposites and the synergistic effects of co-polymers against early blight, leaf spot and stem rot diseases in tomato and potato plants as well as their effects on growth parameters, such as germination percentage, dry mass per plant and root–shoot ratios. Therefore, in the present study Chitosan-Carrageenan nanocomposites were prepared to evaluate their in vitro and in vivo efficacy against the abovementioned diseases in tomato and potato plants and examine fungicide-loaded NP release behavior and the toxicity of NPs in Vero cell lines to assess non-target toxicity.

## 2. Results and Discussion

### 2.1. Nanoparticle Synthesis, Characterization and Optimization

#### 2.1.1. Dynamic Light Scattering

**Figure 1:**
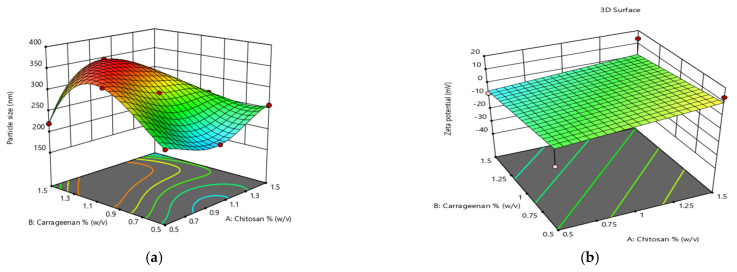
Response surface plot for optimization of chitosan and carrageenan concentration for (**a**) particle size and (**b**) zeta potential by response surface methodology (RSM).

In preliminary experimental trials, the concentrations of chitosan and carrageenan were found to influence the particle size of NPs. The concentrations of chitosan (A), carrageenan (B) and mancozeb (C) were differentiated at three levels, i.e., high (+1), medium (0) and low (−1), to study their effect on particle size. It is apparent from the optimization graph (Figure 1 and Table 1) that particle size increases with an increase in chitosan and carrageenan concentrations. Conversely, the effect is more prominent in the case of chitosan. Zeta potential tends to be more negative with an increase in carrageenan concentration.

**Figure 2:**
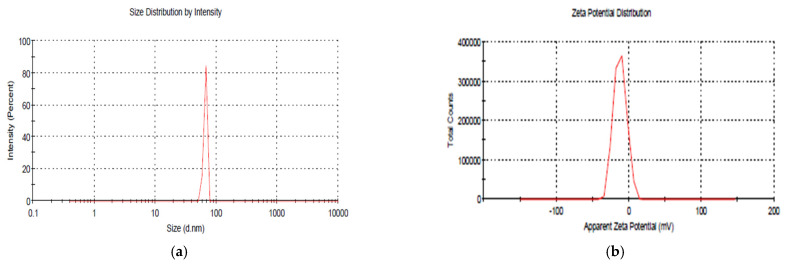
Dynamic light scattering (DLS) analysis of CSCRG nanoparticles. (**a**) Size of blank NPs. (**b**) Zeta potential of blank NPs.

In blank NPs, i.e., without mancozeb loading, the average size was found to be 66.6 nm with a zeta potential of −12.2 mV (Figure 2a,b), while an increased size (231.8 nm) was found in NPs with 1.0 mg/mL mancozeb (CSCRG-1.0). 

For blank nanoparticles, a PDI of 0.553 indicated the synthesis of monodispersed NPs, and values up to ±30 mV for zeta potential are a good indicator of the stability of NPs [3]. Zeta potential values were found to be in the range of +18.1 to −12.2 mV in the present study and thus confirmed the stability of stored nanoparticles (Table 2). Mancozeb-loaded nanoparticles in this study were found to be polydispersed. 

The storage stability of NPs was checked by storing them in distilled water at 4 °C for 20 days. A minor change in size (231.8 nm to 305.6 nm) was found in NPs containing 1.0 mg/mL mancozeb after storing, which may be due to the aggregation of NPs in storage, as dynamic light scattering (DLS) or particle size analyzers work very well for samples that are monodispersed. However, size data often vary in aggregated systems for multiple reasons [30]. Some parameters that affect the size, release profile and stability of the polymeric nanoparticles are the solubility of the drug, the drug-to-polymer ratio, molecular weight, the composition of the polymer, the solvent used for synthesis, pH, homogenization speed and mixing time [31]. A reverse micellar method is often used to obtain stable, small and uniform-sized dispersed nanoparticulate chitosan structures [32]. Earlier, in similar work, while studying nanoparticle properties by taking Chitosan-Carrageenan nanoparticles with cross-linkers (TPP) and their effects on size and the stability of particles, a decrease in size and zeta potential along with an increase in stability were observed due to a change of concentrations [33].

#### 2.1.2. Fourier Transform Infrared Spectroscopy (FTIR)

**Figure 3:**
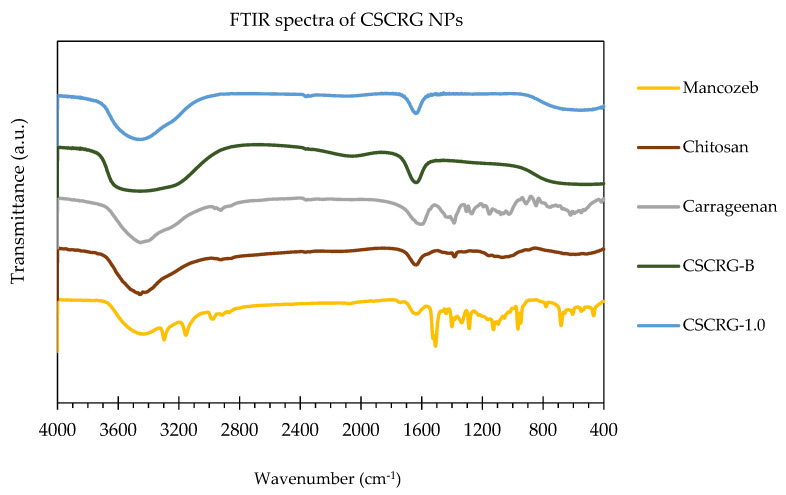
Composite FTIR spectra of mancozeb, blank and loaded CSCRG NPs along with raw polymers.

A strong peak in raw chitosan polymers at 3455.94 cm^−1^ signifies an asymmetrical NH_2_ stretching vibration, while a peak at 1635.75 cm^−1^ denotes an -NH_2_ bond. Bands in raw carrageenan at 1386.31, 1192.17 and 911.20 cm^−1^ are attributed to the presence of S in the polymer. These bands signify the presence of C-O-SO_3_ on C2 of 3,6-anhydrogalactosen, C-O-SO_3_ on C4 of galactose, C-O-SO_3_ on C2 of 3,6-anhydrogalactose and C-O of 3,6-anhydrogalactose, respectively [34]. Strong peaks in blank CSCRG NPs at 3458.03 cm^−1^ signify (associated) asymmetrical NH_2_ stretching vibrations and O-H stretching vibrations. Shifting the peak from 3460.09 cm^−1^ in blank NPs to 3458.03 cm^−1^ in mancozeb-loaded CSCRG NPs signifies the loading of mancozeb in NPs. Protonated chitosan amino groups (–NH_3_^+^) formed a polyelectrolyte complex with the –OSO_3_^−^ of the carrageenan to increase the thermal stability and incorporate a new functionality. The free chitosan amino groups (–NH_2_) were used to form Schiff bases and to covalently encapsulate mancozeb. Moreover, a shift in the percentage transmittance/lump and peaks near 2057 and 1638.12 cm^−1^ in blank NPs to 1638.74 cm^−1^ in mancozeb-loaded NPs indicated mancozeb loading due to the formation of new bonds or amide II N-H deformation and C-N stretching vibration (Figure 3).

When chitosan and carrageenan are mixed, the chemical interactions are affected by their characteristic spectra peaks [35]. The addition of carrageenan caused a shift in absorption bands to 3460.09 cm^−1^, confirming the blending of various components [35]. Some broad bands at 668.70 and 531.12 cm^−1^ in blank and loaded NPs are attributed to the presence of carrageenan in the nanocomposite [36]. The high sulphate content of carrageenan was accountable for peaks in this range [37]. These band shifts and peaks at 1638.74 cm^−1^ might be defended with the robust interactions, perhaps a higher degree of cross-linking [38]. Similarly, Tong et al. [39] found a shift in characteristics peaks of metolachlor from 1672 and 2926 to 1760 and 2883, respectively.

Typical bands of carrageenan, chitosan and mancozeb were identified in the nanoparticle’s spectrum, evidencing the formation of nanoparticles by complexation between the oppositely charged polysaccharides chitosan and carrageenan. 

#### 2.1.3. X-ray Diffraction Spectroscopy (XRD)

**Figure 4:**
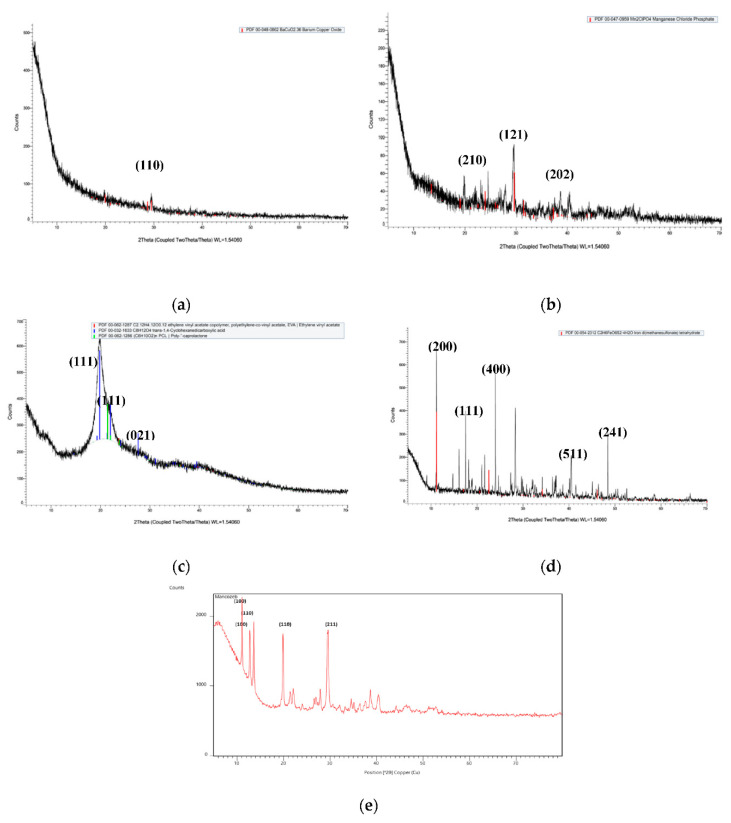
X-ray diffraction spectroscopy plots of (**a**) blank CSCRG nanoparticles, (**b**) mancozeb-loaded CSCRG NPs, (**c**) chitosan, (**d**) carrageenan and (**e**) mancozeb.

Mancozeb showed sharp peaks, as shown in Figure 4, suggesting that it is a highly crystalline material, whereas raw chitosan polymer showed some broad peaks, indicating its semi-crystalline nature. In blank NPs, broad peaks were seen, while in fungicide-loaded NPs, sharp peaks were observed, suggesting mancozeb loading. The sharp crystalline peaks of mancozeb were buried underneath when encapsulated in biopolymeric nanoparticles. This type of pattern was observed by another researcher [40] in XRD patterns for chitosan–dazomet nanoparticles. The sharp peaks at diffraction angles of 19°, 29°, 38° and 41° in loaded nanoparticles matched with the peak pattern of mancozeb, thereby hinting at the encapsulation of mancozeb in Chitosan-Carrageenan nanocomposites.

In similar research, Agnihotri and Aminabhavi [41] developed carvedilol-loaded gellan gum–polyvinyl alcohol microspheres and found intense peaks at 20, 13 and 26°. In carvedilol-loaded NPs, intense peaks were observed between 13 and 26°, indicating the crystalline nature of the drug even after encapsulation. In contrast, Muthukrishnan et al. [42] found thiamine–chitosan nanoparticles to have an amorphous nature while a thiamine–chitosan mixture had a crystalline nature.

#### 2.1.4. Differential Scanning Calorimetry (DSC)

**Figure 5:**
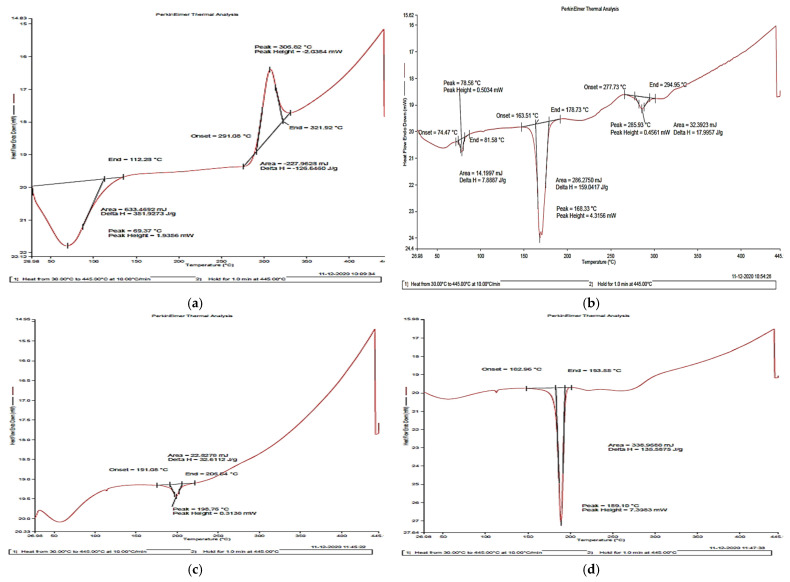

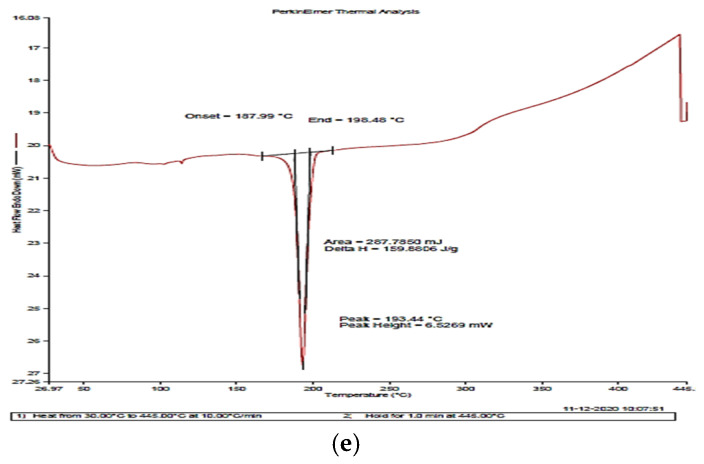
Differential scanning calorimetric thermograms of (**a**) chitosan, (**b**) carrageenan, (**c**) blank CSCRG NPs, (**d**) mancozeb-loaded CSCRG NPs and (**e**) mancozeb.

The DSC thermograms of (a) chitosan, (b) carrageenan, (c) blank NPs, (d) mancozeb-loaded NPs and (e) mancozeb are presented in Figure 5. The melting temperatures (T_m_) of the polymers were calculated. Chitosan showed an endothermic peak between 30 °C and 112 °C, with a peak at 69.37 °C (known as dehydration temperature) due to the loss of water from the hydrophilic group of chitosan. The exothermic peak at 306.82 °C is attributed to the thermal degradation of chitosan. This decomposition was due to monomer dehydration, glycoside bond cleavage and decomposition of the acetyl and deacetylated units of the polymer [43]. The T_m_ peak for carrageenan was obtained at 168.33 °C. The DSC run curve of the blank nanocomposite exhibited a small endothermic peak at about 196.76 °C corresponding to 0.3136 mW of heating. This endothermic peak is attributed to the water evaporation associated with the polymers’ hydrophilic groups [44,45]. Mancozeb-loaded nanoparticles showed a sharp endothermic peak at 189.10 °C, with 7.3983 mW heating and ∆H of 135.56 J/g. Mancozeb showed an endothermic peak at 193.44 °C, with 6.5269 mW of heating and ∆H of 159.88 J/g. These down peaks represent the melting temperature (Tm) [41].

#### 2.1.5. Thermogravimetric Analysis (TGA)

**Figure 6:**
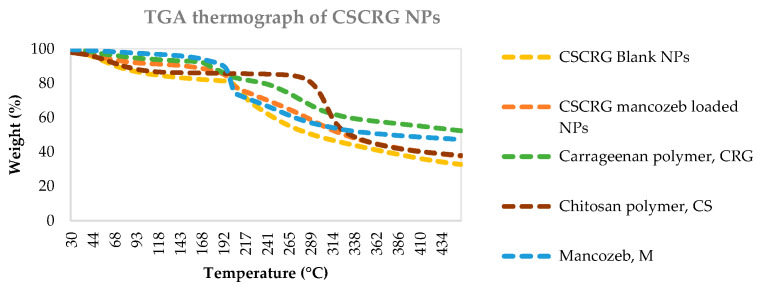
TGA thermograms of blank and mancozeb-loaded nanocomposites along with raw polymers and mancozeb.

Figure 6 shows that the decomposition of mancozeb occurred in two steps with gaseous emissions of SO_2_, H_2_S and CS_2_ at 200 °C. The first weight loss occurs between 30 °C and 200 °C. This weight loss of 24.1% is mainly associated with CS_2_ and H_2_S emissions along with small amounts of SO_2_ and CO. The second weight loss (20.4%) occurred between 200 °C and 300 °C. H_2_S emissions are detected during this decomposition [46]. For chitosan, the first weight loss (9.8%) occurred at 30–120 °C due to water loss, and the second weight loss (45.4%) occurred from 295–350 °C and is assigned to the decomposition of chitosan. For carrageenan, weight loss occurred in three stages: the first at 50–170°, the second at 170–320 °C and the third at 320–450 °C. In a similar study, Sun et al. [47], with their TGA results, revealed that the thermal stability of alginate microbeads was enhanced after alteration with chitosan and k-carrageenan. For blank CSCRG NPs, the first weight loss before 200 °C was attributed to loss of free or chemical-bound water [12,48] and the second occurred from 295–400 °C due to the decomposition of CSCRG NPs, while for loaded NPs the same pattern was observed, with mancozeb and CSCRG NPs decomposing together. The TGA curve for the diclofenac sodium-loaded Chitosan-Carrageenan nanocomposite showed that the third stage (232–310 °C) involved the simultaneous degradation of both drug and polymer [49].

In earlier research, Maluin et al. [40] prepared chitosan–dazomet nanoparticles and found four stages of weight loss. At around 60 °C (the first stage), weight loss occurred due to water loss, while at 245–255 °C (the second stage) it was attributed to the decomposition of chitosan and at the third stage was due to the decomposition of dazomet (332–352 °C). Thus, these nanocompositess were more stable than commercial dazomet. The last stage of weight loss, around 800 °C, was due to char formation. 

#### 2.1.6. SEM/TEM

**Figure 7:**
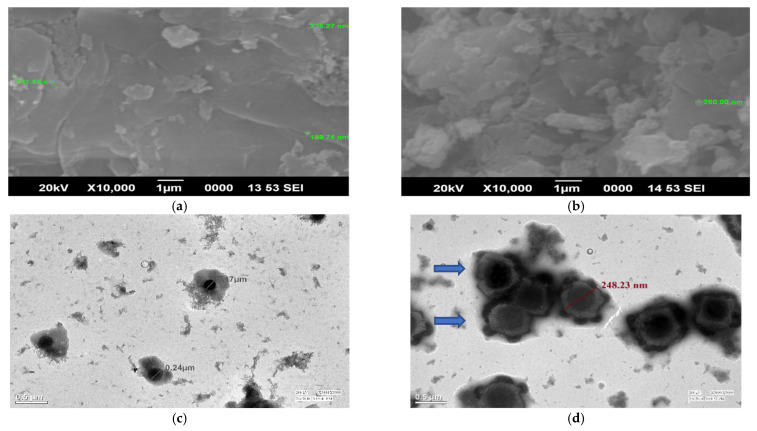
Electron microscopy of blank and loaded nanoparticles. SEM micrographs of (**a**) blank CSCRG NPs and (**b**) mancozeb-loaded CSCRG NPs. TEM micrographs of (**c**) blank CSCRG NPs and (**d**) mancozeb-loaded CSCRG NPs.

Spherical NPs in a size range of 189.74 to 260 nm were observed with SEM (Figure 7a,b). Some porous structures at a high magnification (10,000×) were also seen, which are not seen at lower magnifications (3500×). Earlier, Rampino et al. [50] prepared and observed spherical chitosan-TPP NPs for size and stability evaluations. The spray drying caused particle aggregation, which was clearly visible in the SEM picture of nanoparticles; the nanoparticles were seen to be fused, generating microparticles. Spherical 181 nm (mean size) chitosan nanoparticles, as seen with SEM, were proven to suppress wheat *Fusarium* head blight disease [51]. Another researcher successfully prepared imazethapyr (herbicide)-loaded alginate-alginate-cellulose spherical beads, observed with SEM, for slow herbicide release [52].

TEM micrographs established the synthesis of well-dispersed, small-sized round-shaped nanoparticles with mancozeb seen inside the NPs as a dark grey spot for loaded NPs. Figure 7c,d revealed that blank NPs (170 nm) were spherically shaped without any contrast in the centre. In contrast, mancozeb-loaded NPs (248.23 nm) were spherical and cloverleaf/flower shaped with some granular materials inside and outside the nanoparticles. These spots could be associated with mancozeb molecules encapsulated on the surface and centre region of the NPs [52]. The light contrast between these dark spots and the continuous phase of carrageenan can be attributed to the variance of electronic density between them. Earlier, a scientist synthesized spherical shaped chitosan–TPP nanoparticles in the size range of 30–40 nm, as revealed by TEM [53]. Spherical shaped hexaconazole and dazomet-loaded chitosan NPs of 5.3–156.5 nm in size were observed with HRTEM. An antifungal effect of these NPs on *G. boninense* was observed [32].

### 2.2. In Vitro Study

#### 2.2.1. Antifungal Activity

The nanoformulation and mancozeb were tested for antifungal activity against *A. alternata*, *S. lycopersici*, *A. solani* and *S. sclerotiorum* at three concentrations (0.5, 1.0 and 1.5 ppm) and the results showed that mancozeb-loaded nanoformulations exhibited significant inhibition of mancozeb. The nanoformulation exhibited 100% radial growth inhibition for *S. lycopersici* and *Sclerotinia sclerotiorum* at 1.0 and 1.5 ppm, equivalent to commercial mancozeb at the same concentrations. An increase in the fungal inhibition rate was seen when carbendazim-loaded polymeric nanoparticles were tested against *Fusarium oxysporum* and *Aspergillus parasiticus*, compared to carbendazim alone [5].

From Figure S1 and Table 3, it can be seen that NPs showed maximum inhibition against *Sclerotonia sclerotiorum*, followed by *S. lycopersici* and *A. alternata*. The most negligible inhibition of phytopathogenic fungi by CSCRG NPs was observed in *A. solani*, where blank NPs showed a massive growth of 32.5 ± 3.5 mm with only 50 ± 3.5% inhibition. Mancozeb-loaded NPs at 0.5 and 1.0 ppm also exhibited a mere inhibition of 67.7 ± 1.4% and 67.7 ± 0%, respectively, against *A. solani.* In comparison, at 1.5 ppm (NF1.5), a good inhibition of 83.1 ± 0% was exhibited, comparable to commercial mancozeb (84.6 ± 0%) at this concentration. Copper oxychloride was encapsulated in chitosan–copper oxide/zinc oxide nanoformulations and controlled *Fusarium oxysporum f.* sp. *ciceri* (FOC) in chickpea plants (*Cicer arietinum*) in the same manner [13]. 

In an earlier study, Oh et al. [53] prepared chitosan nanoparticles. They evaluated their in vitro antimicrobial activities against phytopathogens of tomato, namely, *Colletotrichum gelosporidies*, *Phytophthora capsici*, *Sclerotinia sclerotiorum*, *Fusarium oxysporum* and *Gibberella fujikuori.* Maximum inhibitory effects were observed against *F. oxysporum*, followed by *P. capsici*. Similarly, polymeric and solid lipid nanoparticles for persistent release of the conventional fungicides carbendazim and tebuconazole were used with enhanced antifungal activity [54]. Many other researchers have displayed the antifungal efficacy of synthesized nanoparticles [55,56,57].

#### 2.2.2. Encapsulation Efficiency and Loading Capacity

A dose-dependent pattern in the encapsulation efficiency (%) and a non-concentration-dependent pattern in the loading capacity (%) of mancozeb-loaded CSCRG nanoparticles was observed (Table 4). The minimum EE obtained was 17.0 ± 1.20 for the formulations CSCRG-0.5 and a maximum of 58.3 ± 0.83 EE for CSCRG-1.5. A minimum loading capacity of 87.3 ± 0.20% was seen in CSCRG-0.5, which might be due to minimal mancozeb in the sample. Maximum LC (95.5 ± 1.15) was found for NPs containing 1.0 mg/mL mancozeb with optimum mancozeb available for loading (Table 4). The LC in our case was found to be higher compared to vincristine-loaded folic acid–chitosan conjugated nanoparticles synthesized by another researcher [58], signifying a good ionic interaction between the fungicides and the nanoparticles. Earlier, acetamiprid-loaded alginate–chitosan nanocapsules were prepared using the ionic pregelation and polyelectrolyte complexation method. Due to ionic contacts between positively charged ammonium groups of chitosan and negatively charged carboxylate groups of alginate, a polyionic complex was formed with a loading capacity of 62% [3]. Here, in this study, two oppositely charged polymers were used and the loading capacity was further enhanced by the addition of TPP. Similarly, a flash nanoprecipitation technique was used to produce abamectin-loaded nanoparticle with a loading capacity > 40% and an encapsulation efficiency >95% using amphiphilic polymers PLGA-b-PEG [59]. 

#### 2.2.3. Release Behavior

**Figure 8:**
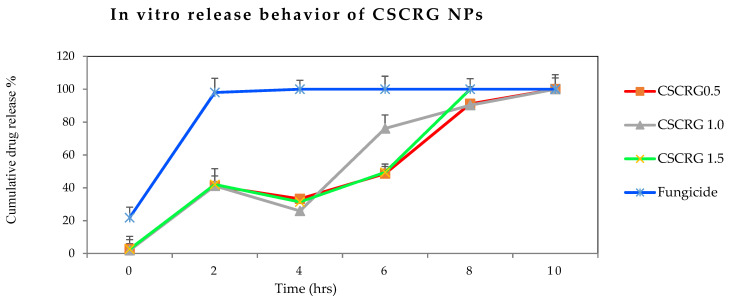
In vitro release behavior showing the slow and sustained release of mancozeb from Chitosan-Carrageenan (CSCRG) conjugated nanoparticles in phosphate-buffered saline (pH 7.2).

In Figure 8 it can be seen that 98% of the commercial drug was released within 2 h, while the percentage at this point was 42% for the three mancozeb-loaded nanoformulations. A release of 76% was observed for CSCRG-1.0 after 6 h, while the other two mancozeb-loaded nanoformulations exhibited a 49% cumulative release percentage for the same period. Maluin et al. [32] achieved a sustained release of 99.91% with a prolonged time of 86 h when he encapsulated commercial hexaconazole in chitosan NPs using the ionic gelation method. Using the same synthesis method and the same biopolymer but with different agrochemicals (spinosad and permethrin), Sharma et al. [60] achieved 30% (spinosad) and 75% (permethrin) release within 5 h from chitosan nanoparticles. However, the most prolonged release mechanisms were exhibited in our study by CSCRG-0.5 (NPs containing 0.5 mg/mL mancozeb), which showed a total release of mancozeb in 10 h in the buffer. Therefore, it can be said that 0.5 mg/mL is the optimal concentration for sustained release and that higher doses may not be entrapped adequately by the polymer matrix nanosystem. 

In an earlier study, polymeric and solid lipid nanoparticles were used by Campos et al. for persistent release of the conventional fungicides carbendazim and tebuconazole [55]. Polymeric nanoparticles released about 47% of the fungicide, while the solid lipid nanoparticles released nearby 51% in six days. A controlled release nanoformulation of another low water-soluble insecticide, acetamipirid, was developed by Kumar et al. [3]. Release studies in diverse soil and media improved the controlled release compared to commercial acetamipirid and acidic soils gave the best results. A detailed study of the controlled release of the herbicide Tebuthiuron encapsulated in microparticles of calcium alginate and its correlation with leaching depth using bioindicator plants was demonstrated [61]. Rychter [62] found a herbicidal effect exhibited by the herbicide glyphosate with chitosan nanoformulations and established the slow and sustained release of the active ingredient from the polymer matrix. A slow and incomplete release of carbendazim from chitosan–pectin nanoparticles into the media at different pH levels was observed by another researcher [19]. The average cumulative percentage release of carbendazim from the nanoformulation was found to be 61.9 ± 0.1% at pH 4.0, 50.4 ± 0.13% at pH 7.4 and 62.8 ± 0.13% at pH 10.0 after 48 h, while for pure carbendazim the percentage release was 82.4 ± 17% at pH 4.0, 67.7 ± 0.1% at pH 7.4 and 86.8 ± 0.2% at pH 10.0. These data confirmed persistent release in the carbendazim nanoformulation [19]. The great advantage of the Chitosan-Carrageenan formulation is that chitosan dissolved in a water solution of mancozeb may play a double role—as an environmentally friendly adjuvant (sticker) and as a polymeric carrier for prolonged release of mancozeb without causing any harm to non-target organisms due to the food-grade quality of both the biopolymers.

### 2.3. In Vivo Study

#### 2.3.1. Bioefficacy of Nanoparticles in Pot House Conditions

All the mancozeb-loaded NPs showed a disease control efficacy (DCE%) percentage of 70.3 ± 6.9, 73 ± 3.6, 79.4 ± 1.7 and 77.2 ± 2.7%, respectively, which is relatively high compared to commercial mancozeb, the efficacy of which against plant pathogenic fungi *A. alternata*, *S. lycopersici*, *A. solani* and *S. sclerotiorum* was 76.6 ± 5.8, 69.5 ± 1.8, 76 ± 1.1 and 68 ± 6.9%, respectively. It is evident from Figure S2 and Table 5, that the nanoformulation showed an enhanced DCE% for all the four phytopathogenic fungi of tomato and potato except *A. alternata.*

#### 2.3.2. Effect of Treatment on Germination Percentage

**Figure 9:**
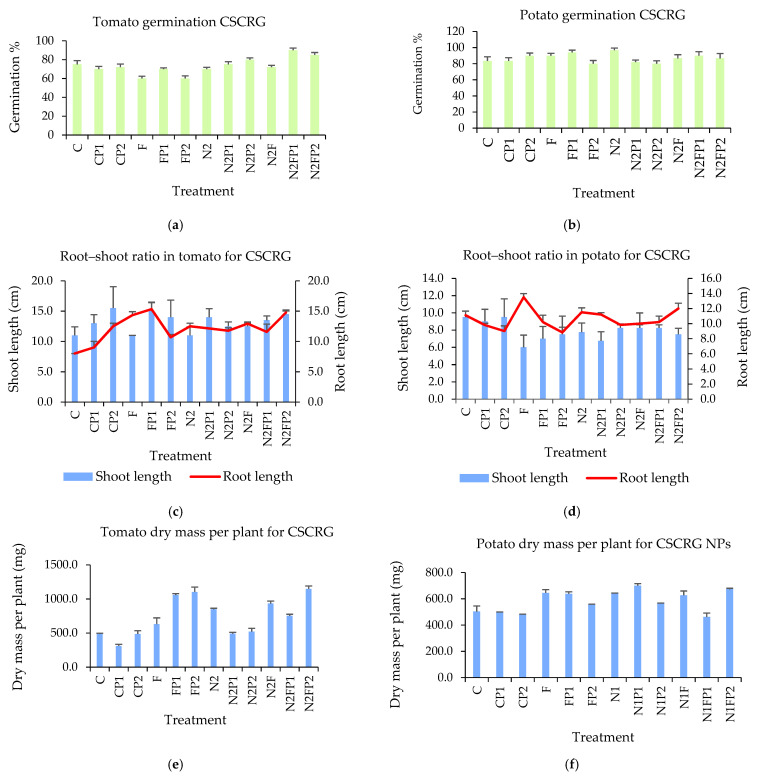
In vivo effects of CSCRG NP treatments on tomato and potato plant growth parameters: (**a**,**b**) germination percentage, (**c**,**d**) root–shoot ratio and (**e**,**f**) dry mass per plant. C—control (No treatment), CP1—plants made sick with pathogen P1, F—plants treated with commercial fungicide, FP1—plants made sick with the pathogen and treated with commercial fungicide, N1—blank NP treatment, N1P1—plants made sick with pathogen and treated with blank NPs, N1F—plants treated with fungicide-loaded NPs, N1FP1—plants made sick with pathogens and treated with fungicide loaded NPs, CP2—plants made sick with pathogen P2, FP2—fungicide-treated plants made sick with pathogen P2, N1P2—plants made sick with pathogen P2 and treated with blank NPs, N1FP2—plants made sick with pathogen P2 and treated with fungicide loaded NPs. For tomato: Pathogen P1 = *A. alternata*, P2 = *S. lycopersici*; for potato: Pathogen P1 = *A. solani*, P2 = *Sclerotinia sclerotiorum*.

The in vivo study showed that germination of the tomato seeds treated with the nanoformulation was 70 and 72% for blank and loaded NPs, while the seeds treated with mancozeb showed germination of 60% in comparison to nanoformulation-treated seeds. In potato plants, the germination percentage was 97% for blank NPs and 87% for loaded NPs, and 90% for commercial mancozeb, as shown in Figure 9a,b.

#### 2.3.3. Effect of Treatment on Dry Weight per Plant

The in vivo study showed that dry mass (mg) of the tomato seeds treated with the nanoformulation was 843.3 mg and 932.5 mg for blank and loaded NPs, respectively, while the seeds treated with mancozeb exhibited a dry mass of 630 mg in comparison to nanoformulation-treated seeds. In potato plants, dry weight was 803.3 mg for blank NPs and 785 mg for loaded NPs, respectively, and 645 mg for plants treated with commercial mancozeb. An effective decline was observed in germination (%) and dry mass (mg) by pot house experiment in mancozeb-treated seeds compared to the seeds treated with the nanoformulation.

#### 2.3.4. Effect of Treatment on the Root–Shoot Ratios of Plants

The average root length of tomato plants was 12.5 and 12.9 cm for blank and mancozeb-loaded CSCRG-1.0 NPs, respectively, and a greater length of 14.3 cm was recorded for commercial mancozeb. Similarly, in potato plants, root length was found to be 11.5 and 10 cm in blank and mancozeb-loaded CSCRG-1.0 NPs, respectively, a greater length of 13.5 cm recorded for commercial mancozeb. The average shoot length of tomato plants was 11 and 12.5 cm for blank and mancozeb-loaded CSCRG-1.0 NPs, respectively, and a decreased length of 10 cm was recorded for commercial mancozeb. Similarly, in potato plants, shoot length was found to be 7.8 and 8.3 cm in blank and mancozeb-loaded CSCRG-1.0 NPs, respectively, a decreased length of 6 cm being recorded for commercial mancozeb. An opposite pattern was thus observed for root and shoot length with nanoformulation and mancozeb treatments. The overall effect of the nanoformulation on plant growth parameters (germination percentage, root–shoot ratio and dry biomass of tomato and potato plants) is shown in Figure 9a–f.

In conclusion, seeds or plants treated with the nanoformulation showed higher growth than the seeds/plants treated with mancozeb. These results confirmed that the nanoformulation is less harmful than mancozeb to plants in terms of plant growth parameters (germination percentage, root–shoot ratio and dry biomass/weight).

In earlier similar work, pyraclostrobin, a low water-soluble fungicide, was loaded onto chitosan–lactide co-polymer nanoparticles at different concentrations. It was found that the nanofungicide was either similar to or less efficient at preventing inhibition of *C. gossypii* compared to commercial pyraclostrobin after three days post application. However, an increase in inhibition was observed after seven days post application compared to application with the active ingredient alone [63]. In another trial, kaempferol (another low water-soluble fungicide) loaded onto lecithin–chitosan displayed 67% inhibition efficacy after 60 days against *Fusarium oxysporum* in a Petri dish [64]. An increase in the fungal inhibition rate was observed with carbendazim-loaded polymeric nanoparticles when tested against *Fusarium oxysporum* and *Aspergillus parasiticus* compared to carbendazim alone [65]. Rychter [62] found that a high concentration of chitosan nanoparticles with encapsulated glyphosate caused a reduction in toxic effects against roots of *Avena sativa* and *Raphanus sativus.*

### 2.4. Cytotoxicity of Nanoformulations in Vero Cell Cultures

**Figure 10:**
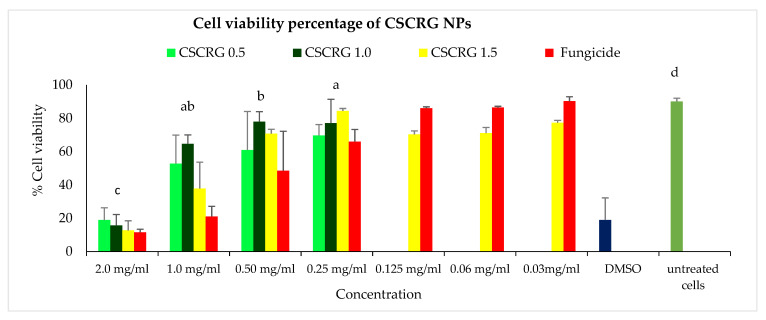
Toxicity of CSCRG NPs in the form of percentage cell viability in Vero cell lines. Caps on bars represent standard deviation. Different letters in graph columns show significance levels at *p* ≤ 0.05 according to a Bonferroni post hoc *t*-test.

The highest cell viability of 84.39% was obtained for the CSCRG-1.5 formulation, followed by CSCRG-1.0 (77.05%) at a 0.25 mg/mL concentration, comparable to untreated cells (90.11%). As we decreased concentrations of the treatment, cell viability increased. The toxicity of the commercial formulation and the nanoformulation of hexaconazole on Vero cell lines increased with increasing pesticide concentration, i.e., from 10 to 20 ppm. The blank nanoformulation did not show any cytotoxicity because the polymers used to prepare the nanocapsules are biocompatible [20]. The lowest cell viability (12.70%) was seen in CSCRG-1.5, followed by CSCRG-1.0 (15.68%) at 2.0 mg/mL concentration of NPs. Commercial mancozeb also showed almost the same cell viability (11.56%) at this concentration (Figure 10). Positive control DMSO, which is toxic to cells, also exhibited cell viability of 18.98%. From the above results, we can conclude that the optimum dose of concentration is 0.25 mg/mL for cell viability with reduced cell cytotoxicity.

The cytotoxicity of the herbicide (metsulfuron-methyl)-loaded pectin nanoparticles was evaluated by Kumar et al. [5] using healthy cell lines (Vero cell lines) and compared with commercial herbicide. An in-field evaluation of the *Chenopodium album* plant was performed using a pectin nanocarrier. The results showed that the application of herbicide-loaded nanoparticles could reduce the use of herbicides with improved efficacy and environmental safety [5]. The results in the present study are in accordance with a previous study in which the encapsulated herbicide paraquat was found to be less toxic to alveolar and mouth cell lines compared to the trade form. The toxicity of NPs on A549 cell lines was comparatively low comapred to the KB cell lines for the mainstream doses [65,66]. Therefore, in conclusion, we can say that nanoparticles decreased the cytotoxicity and genotoxicity of the pesticide for non-target organisms, thereby offering a greener approach to combat diseases in agricultural crops. 

## 3. Materials and Methods

### 3.1. Reagents, Fungal Isolates and Plant Materials

Chitosan (degree of deacetylation ≥ 75%) and sodium tripolyphosphate (TPP) was purchased from Sigma-Aldrich, St. Louis, MI, USA. Carrageenan was taken from Hi-media, India. From the local market we purchased the fungicide mancozeb (RidomilGold^®^, Syngenta, Basel, Switzerland) with mancozeb as the active ingredient (a.i.) 64.0% *w*/*w*. The Vero cell lines used in the cytotoxicity study was maintained at National Research Center on Equines, Hisar, India. Tomato (Arun Hisar/Sel-7) and potato (Kufri Pukhraj) seeds were purchased from the vegetable division of Chaudhary Charan Singh Haryana Agriculture University (CCSHAU, Hisar, India). A glass house situated in Chitrakoot belonging to the university was used for the in vivo pot experiment. Pathogenic fungi (*Alternaria solani*, *A. alternata*, *Stemplyleum lycopersici* and *Sclerotinia sclerotiorum*) used in the study were purchased from Indian Type Culture Collection, IARI, New Delhi. The cultures were revived as per catalogue instructions.

### 3.2. Synthesis of Blank and Mancozeb-Loaded Chitosan-Carrageenan Conjugated Nanoparticles

Nanoparticles were synthesized following Chopra et al. [67] with some modifications. A stock solution of 1.0 mg/mL chitosan in 1% glacial acetic acid (*v*/*v*) was prepared, continuously stirred with a magnetic stirrer for one hour and stored overnight at room temperature to dissolve completely. Different amounts of mancozeb, i.e., 20, 40, 60 mg, in solid form, were taken in three conical flasks of 100 mL capacity to make the final concentrations of 0.5, 1.0 and 1.5 mg/mL mancozeb in solution, respectively. To each flask 20 mL stock solution of chitosan was added and stirred for 10 min. NPs were prepared dropwise by adding 20 mL of 1.5 mg/mL carrageenan (pre-warmed at 70 °C in a water bath for 20 min) to each flask followed by continued magnetic stirring for 30 min. Then, 2 mL TPP (1%) was added dropwise with continuous stirring of the solution for 45 min. After that, 10 µL Tween-20 (used as a surfactant and stabilizing agent) was added to each flask and the contents were stirred for another 45 min. The suspension was centrifuged at 12,000 rpm for twenty minutes, after which the pellets were washed with 10 mL distilled water and centrifuged at the same rpm. The pellets thus obtained were stored at 4 °C in a 1.5 mL centrifuge vial and used for further study. Blank nanoparticles were synthesized following all the above steps except the addition of mancozeb.

### 3.3. Characterization

#### 3.3.1. Size Optimization, Polydispersity Index and Zeta Potential

Design-Expert Software (Version 13, Stat-Ease Inc., Minneapolis, MN, USA) was used to design experiments and statistical analysis was conducted to select the optimum formulation variables and ingredient concentrations. The optimization of mancozeb-loaded chitosan-carrageenan NPs was carried out using a central composite design with a sum equal to 1 as per standard protocol. Three factors, i.e., the concentrations of chitosan, carrageenan and mancozeb, were varied, and TPP concentration was maintained as a constant. Particle size and zeta potential were chosen as the response variables.

The synthesized NPs were characterized by dynamic light scattering (DLS) using a Zetasizer Nano ZS90 (Malvern Instrumentations, Holtsville, UK). For this purpose, 50 µL NPs were poured in a disposable cuvette and dispersed in 950 µL double-distilled water, and percentage intensity was measured at 25 °C.

#### 3.3.2. Fourier Transform Infra-Red (FTIR) Spectroscopy

Fourier transform infra-red (FTIR) spectroscopy was performed with a fine powder of mancozeb, blank, and mancozeb-loaded NPs and polymers (5.0 mg each). FTIR spectra were recorded using potassium bromide (KBr) in a 1:10 ratio using an AVATAR 370 FTIR spectrometer (Therma Nicolet spectrometer, San Jose, CA, USA) at room temperature in a scan range between 4000 and 400 cm^−1^ with a resolution of 4 cm^−1^. Graph peaks was used to determine the ionic interactions between the test fungicide, polymers and NPs to confirm mancozeb loading. Data were analyzed using the online website Spectroscopic Tools, 2019 (St. Thomas “Spectroscopic Tools” URL: http://www.science-and-fun.de/tools/, Accessed on 30 November 2021).

#### 3.3.3. Transmission Electron Microscopy (TEM)

Loading of mancozeb by the polymeric NPs was further confirmed with the Tecnai™ (Thermo Fisher Scientific, Waltham, MA, USA) TEM. Samples were sonicated before analysis and were prepared by adding a drop of an aqueous solution of nanoparticles on a carbon-coated copper grid followed by air drying at room temperature. The TEM images were taken at an operating voltage of 200 kV.

#### 3.3.4. Scanning Electron Microscopy (SEM)

A 10 mg finely powdered sample (lyophilized) was used for analysis, and a JEOL Model JSM-6390LV (JEOL Ltd., Tokyo, Japan) obtained SEM micrographs at an operating voltage of 20 kV. A dry powder sample was placed onto an aluminium specimen stub covered with a double-sided carbon adhesive disc and sputter-coated with gold (20 kV for 3 min).

#### 3.3.5. X-ray Diffraction Spectroscopy (XRD)

XRD measurement of the mancozeb powder was accomplished using a D-8 Advance Diffractometer (Bruker AXS, Karlsruhe, Germany) in a step scan mode with a tube voltage of 40 kV and the current set at 40 mA. The samples were scanned in the 2θ range of 5–40°.

#### 3.3.6. Thermal Analysis Using Differential Scanning Calorimetry (DSC)

The thermal profiles of mancozeb, blank and mancozeb-loaded polymeric nanoparticles were determined using the CIF facility at LPU, Jalandhar by differential scanning calorimetry (DSC 4000 System, Perkin Elmer, Waltham, MA, USA). Samples containing 3 mg were weighed accurately on standard nickel–chromium sample plates using an empty pan as a reference, an alumina-coated aluminium furnace used in the instrument for heating purposes. DSC scans were recorded at a heating and cooling rate of 10 °C/min. The samples were heated from 30–445 °C and held for 1 min at 445 °C. Pure (99.99%) nitrogen gas was liquidated into the system at a 20 mL/min flow rate to maintain an inert atmosphere.

#### 3.3.7. Thermogravimetric Analysis (TGA) 

A Thermo Gravimetric Analyser (TGA 4000, Perkin Elmer, Billerica, MA, USA) was used for TGA at LPU, Jalandhar. The instrument was operated at a heating rate of 10 °C/min from 30 to 445 °C and held for 1 min at 445 °C. Pure nitrogen gas was introduced into the system at a flow rate of 20 mL/min to maintain an inert atmosphere in the system.

#### 3.3.8. Encapsulation Efficiency (%) and Loading Capacity (%)

To determine the encapsulation efficiency (%), mancozeb-loaded polymeric nanoparticles were centrifuged at 15,000 rpm for 35 min. The supernatant was collected in a clean, sterile Eppendorf tube and analyzed for UV–Vis spectra at 290 nm. The content of free mancozeb in the supernatant was determined with a UV–Vis spectrophotometer (NanoDrop2000c, Thermo Fisher Scientific, Wilmington, DE, USA) at 290 nm using the supernatant of their corresponding blank nanoparticles without loaded drugs as the basic correction. The encapsulation efficiency (%) was calculated with the help of the following equation:

$$\mathrm{Encapsulation}\;\mathrm{efficiency}\;\left(\%\right)=\frac{\mathrm{Mancozeb}\left(\mathrm{total}\right)-\mathrm{Mancozeb}\left(\mathrm{free}\right)}{\mathrm{Mancozeb}\left(\mathrm{total}\right)}\times 100$$

Loading capacity was calculated with the following equation:

$$\mathrm{Loading}\;\mathrm{capacity}\;\left(\%\right)=\frac{\mathrm{Mass}\;\mathrm{of}\;\mathrm{mancozeb}\;\mathrm{in}\;\mathrm{polymeric}\;\mathrm{nanoparticles}}{\mathrm{Mass}\;\mathrm{of}\;\mathrm{polymeric}\;\mathrm{nanoparticles}\;\mathrm{recoverd}}\times 100$$

### 3.4. In Vitro Study

#### 3.4.1. Antimicrobial Activity

For the in vitro study, the effect of NPs on mycelial growth of selected pathogen strains was carried out on potato dextrose agar (PDA, 2%) media using the mycelium-inhibition method described by Kumar et al. [19] with some modification. The poisoned food technique was used to determine antifungal activity. Different concentrations (0.5, 1.0 and 1.5 ppm) of various nanoparticles in aqueous solution were used in antifungal activity tests against four fungus species, viz. *A. alternata*, *A. solani*, *S. lycopersici* and *S. sclerotiorum*. The stock solution at a concentration of 100 mg/l was prepared in distilled water for mancozeb, the mancozeb-loaded nanoformulation, and the nanoformulation without mancozeb. Then, 0.5, 1.0 and 1.5 mL were added to conical flasks containing autoclaved potato dextrose agar media (HiMedia, Mumbai, India; liquid; temperature, 40 °C) to obtain a final volume of 100 mL with required concentrations. Then, the media was poured into sterile Petri dishes (90 mm × 15 mm) with the ppm mentioned above for the various formulations which were kept separate and allowed to solidify. Mycelial discs of uniform size (diameter, 5.0 mm) were taken from the peripheral end of 7-day-old cultures of test pathogens and placed in the centre of test Petri dishes. All the Petri dishes were incubated at 28 ± 1 °C for 7 days, and observations of radial mycelial growth were recorded after 4 days. All the treatments were replicated three times and repeated twice. The inoculated plates were compared with controls (without nanoparticles) to calculate the percentage inhibition rate of mycelia of the pathogen using the formula:

% Inhibition rate = Mc − Mt/Mc × 100

where Mc is the mycelial growth in the control and Mt is the mycelial growth in treated samples.

#### 3.4.2. Controlled Release Behavior

Dialysis tubing (Himedia, molecular weight cut off 12,000–14,000 Da) was used to assess the in vitro release behavior of mancozeb from polymeric nanoparticles at 37 °C from 2–24 h. Lyophilized and sonicated 10 mg nanoparticles were supplemented with 1 mL sterile phosphate-buffered saline, PBS (pH 7.0), dispensed in a dialysis membrane with closed clips at one end, using a sterile pipette, and immersed in 10 mL of the same sterile PBS (pH 7.0) in different beakers of 20 mL capacity. All the beakers were incubated in a laboratory shaker at 160 rpm at 37 °C. After fixed intervals of time (30 min), 1 mL of phosphate-buffered saline (pH 7.0) was pipetted out from the beaker, and the same amount of fresh buffer was added to each beaker to maintain the sink condition. The absorbance of the resulting solution was measured at 260 nm (Nanodrop 2000c, Thermo Fisher Scientific, Waltham, MA, USA) to determine the concentration of mancozeb in the buffer with the help of a standard curve. The cumulative release percentage was plotted against time.

### 3.5. In Vivo Study

#### 3.5.1. Bioefficacy of Nanofungicide against Target Fungi in Pot House Conditions

The bioefficacy of different polymeric nanoparticles in controlling early blight and stem rot (or southern blight) in potato (*Solanum tuberosum* L.) and early blight and leaf spot in tomato (*Lycopersicon esculentum* L.) were studied in pot house conditions in a glass house under natural light and temperature. Pots were filled with sandy soil from the adjacent area of Jannayak Chaudhary Devilal, Vidyapeeth, Sirsa. NP-coated seeds (5 seeds/pot for tomato and 3 seeds/pot for potato) were sown in pots filled with soil (pH 7.7; temperature, 20 °C) already made sick with pathogenic fungi two days before sowing.

##### Treatment

The seeds were first thoroughly washed with double-distilled water to remove any dust or other particles. Then, seeds were treated with 4% sodium hypochlorite (surface disinfectant) for 10 min and thoroughly washed 2 times with distilled water. The seeds were then dipped in carboxymethyl cellulose (5 gm in 100 mL distilled water) for 10 min and allowed to dry for 10 min at room temperature. The seeds were treated with different nanoparticles (10 ppm) for 2.5 h and allowed to dry at room temperature. Five seeds/pot of tomatoes and 3 seeds/pot of potatoes were sown at 1.0 cm depth with the help of sterile forceps, with an equal gap between seeds, and watered regularly.

To initiate early blight and leaf spot diseases in tomato and potato plants, respectively, 40-day-old plant parts were made sick by spraying them (15 mL/plant) with an aqueous conidial suspension (3.1 × 10^7^ CFU/mL) of the respective pathogens. The plants were then covered with transparent plastic bags for five days to maintain the humidity required for the disease outbreak. Foliar spray of polymeric NPs (10 ppm, 15 mL/plant) was performed after the disease outbreak to assess the bioefficacy of NPs against the disease. For stem rot disease outbreak, the conidial spores were kept at the root–shoot junction of potato plants, as it is at this junction that the disease breaks out. Commercial fungicide mancozeb (Ridomil Gold) was used as a positive control for both plants.

##### Disease Assessment

Leaves from each replicate were randomly selected for recording disease data. In stem rot, the surface area of the diseased stem was observed to determine the disease severity. The progress of the disease was observed for 10 days, and disease severity was recorded using a standard rating of 0–5.

Grades of disease severity:

0 = 0% leaf/stem area infected, 1 = 1–5% leaf/stem area infected, 2 = 6–10% leaf/stem area infected, 3 = 11–20% leaf/stem area infected, 4 = 21–30% leaf/stem area infected, 5 = more than 31% leaf/stem area infected.

$$\mathrm{Disease}\;\mathrm{severity}=\frac{\mathrm{Sum}\;\mathrm{of}\;\mathrm{all}\;\mathrm{individual}\;\mathrm{disease}\;\mathrm{rating}}{\mathrm{Total}\;\mathrm{number}\;\mathrm{of}\;\mathrm{leaf}\;\mathrm{assessed}\;–\;\mathrm{maximum}\;\mathrm{rating}\;}\times 100$$

Disease control efficacy (%) was calculated using the formula of Kaur et al. [14].

$$\mathrm{DCE}\;(\mathrm{in}\%)=\frac{\mathrm{Disease}\;\mathrm{severity}\;\mathrm{in}\;\mathrm{control}\;-\;\mathrm{Disease}\;\mathrm{severity}\;\mathrm{in}\;\mathrm{treatment}}{\mathrm{disease}\;\mathrm{severity}\;\mathrm{in}\;\mathrm{control}\;}\;\times \;100$$

Bioefficacy was also noted in leaf area and plant growth parameters, such as plant height, root–shoot ratio and plant dry weight, to assess the overall health and vigor of the test plants.

##### Effects of Treatment on Plant Growth Parameters

Germination percentage was noted after 28 days of sowing with 3–4 true leaves. Root and shoot length were recorded after harvesting with a meter scale from the root–shoot junction. For dry biomass, potato and tomato fruits were excised from stolons, and the remaining parts of the plants (3 plants) put in brown envelopes and air-dried in an oven at 40 °C for 7 days.

### 3.6. Cytotoxicity of Nanoparticles in Vero Cells

The cytotoxic activity of nanoparticles was assessed by colorimetric assay using resazurin dye (7-Hydroxy-3H-phenoxazin-3-one 10-oxide, Hi Media), blue in color and non-fluorescent until it reduced to the highly fluorescent pink colored resorufin in response to the chemical reduction of the growth medium resulting from cell growth. Continued cell growth maintains a reduced environment, while inhibition of growth maintains an oxidized environment. Reduction related to growth causes the REDOX indicator to change from the oxidized (non-fluorescent, purple color) form to the reduced (fluorescent, pink color) form. 

Protocol:

- Briefly, Vero cell lines (derived from the kidney of an African green monkey) at a density of 1 × 10^4^ per well were cultured in a 100 μL volume of cell culture medium (EMEM supplemented with 10% fetal bovine serum and antibiotics) in a 96-well cell culture plate.
- The cultured cells at 70–80% confluency were treated with different concentrations of nanoparticles (0.25–2000 μg/mL), well dispersed in 100 μL of deionized water with sonication and incubated in a CO_2_ incubator (at 37 °C with 5% CO_2_) for 24 h.
- After incubation, the samples were treated with 10 μL of the resazurin dye (1 mg/mL) prepared in EMEM media and incubated for 4 h under the same conditions.
- After 4 h, the pink-coloured resorufin is formed and absorbance was observed by a spectrophotometer (ELISA plate reader) at 590 nm.

The percentage reduction of blue to pink colour is directly proportional to cell viability. With the help of absorbance measures, cell viability/cytotoxicity was calculated, dead cells being responsible for high absorbance. 

### 3.7. Statistical Analysis

All the results values are shown as mean ± standard deviation. Comparison between data was made using one-way analysis of variance (ANOVA) and a *t*-test with a *p*-value ≤ 0.05 as the minimal significance level.

## 4. Conclusions

The fungicide mancozeb was encapsulated in biopolymers (chitosan and carrageenan) to form a nanomatrix using the ionic gelation method. The prepared nanoparticles were characterized by various spectroscopic (FTIR, XRD) and microscopic (SEM, TEM) techniques to confirm their synthesis. The average size of nanoparticles in DLS was 66.6–231.82 nm. DSC and TGA established the transition and thermal stability of synthesized NPs. In vitro antifungal activity of synthesized NPs was found to be comparable to commercial fungicide, i.e., mancozeb. In the pot house experiment, comparable disease control efficiency (DCE %) was exhibited by nanoformulations in both tomato and potato plants. The higher loading capacity may explain this observation. Other plant growth parameters showed comparable results to commercial mancozeb. The resazurin toxicity assay on a Vero cell lines confirmed good cell viability up to 0.5 mg/mL and the nanoformulation was found to be less toxic than mancozeb. Sustained and prolonged release behavior for nanoformulations has been recorded, which may be due to their high surface area. Hence, the results of the study support the utilization of biopolymeric nanoparticles for sustainable crop production with minimized pollution and toxicity to non-target organisms.

## Figures and Tables

**Table 1:** Experimental runs for optimization of mancozeb-loaded chitosan-carrageenan NPs.

Run	Factor 1	Factor 2	Factor 3	Response 1	Response 2
	Concentrations			Particle Size (nm)	Zeta Potential (mV)
	A: Chitosan (*w/v*)	B: Carrageenan (*w/v*)	C: Mancozeb (*w/v*)		
1.	1	1	0.5	181.76	−10.92
2.	1.5	1.5	1.5	258.5	−12.2
3.	0.5	0.5	0.5	220.2	−10.7
4.	0.5	1	1	66.6	−9.87
5.	1	1.5	1	332.4	−10.7
6.	1.5	0.5	1.5	346.2	−9.06
7.	0.5	1.5	1.5	338.7	−7.59
8.	1.5	1.5	0.5	231.8	13.9
9.	0.5	0.5	1.5	261	14.9
10.	1	1	1	202.4	16
11.	1	1	1.5	413.3	7.97
12.	1	1	1	434.4	6.86
13.	1.5	1	1	305.6	11.5
14.	1	0.5	1	359.9	6.39
15.	1.5	0.5	0.5	590.8	13.3
16.	1	1	1	436.5	12.6
17.	0.5	1.5	0.5	105.6	−7.59

**Table 2:** Size, zeta potential and PDIs of freshly prepared and stored blank and mancozeb-loaded CSCRG NPs.

Freshly Prepared Nanoparticles			
Nanoparticles	Size	Zeta Potential	PDI
Blank Chitosan-Carrageenan nanocomposite (CSCRG-B)	66.6 ± 7.5	−12.2 ± 1.2	0.553 ± 0.1
Mancozeb (1.0 mg/mL)-loaded Chitosan-Carrageenan nanocomposite (CSCRG-1.0)	231.8 ± 0.9	13.9 ± 2.4	1 ± 0.2
Storage stability of CSCRG NPs at 4 °C after 20 days			
Blank Chitosan-Carrageenan nanocomposite (CSCRG-B)	252.0 ± 2.1	18.1 ± 2.8	0.562 ± 0.2
Mancozeb (1.0 mg/mL)-loaded Chitosan-Carrageenan nanocomposite (CSCRG-1.0)	305.6 ± 0.7	7.97 ± 1.4	1 ± 0.5

Mean ± SD; three replicates were kept of each treatment.

**Table 3:** In vitro antifungal activity of blank and mancozeb-loaded (1.0 mg/mL) CSCRG NPs.

Phytopathogenic Fungus	Treatment	CSCRG NPs Fungus Diameter (mm)	CSCRG NPs % Inhibition = dc − dt/dc × 100	Mancozeb Fungus Diameter (mm)	Mancozeb % Inhibition = dc − dt/dc × 100
*A. alternata* (ITCC3640)	Blank NPs N 1.0 (1.0 ppm)	12.5 ± 0.7	83.9 ± 0.7 b	--	--
	Loaded NPs NF 0.5 (0.5 ppm)	19.5 ± 0.7	74.8 ± 0.7 bc	12 ± 1.4	84.5 ± 1.4 b
	Loaded NPs NF 1.0 (1.0 ppm)	14 ± 0	81.9 ± 0 b	11.5 ± 0.7	85.2 ± 0.7 b
	Loaded NPs NF 1.5 (1.5 ppm)	13.5 ± 0.7	82.6 ± 0.7 b	10.5 ± 0.7	86.5 ± 0.7 b
*S. lycopersici* (ITCC5431)	Blank NPs N 1.0 (1.0 ppm)	12.5 ± 0.7	62.1 ± 0.7 c	--	--
	Loaded NPs NF 0.5 (0.5 ppm)	14.5 ± 0.7	56.1 ± 0.7 c	14.5 ± 0.7	56.1 ± 0.7 c
	Loaded NPs NF 1.0 (1.0 ppm)	0 ± 0	100 ± 0 a	0 ± 0	100.0 a
	Loaded NPs NF 1.5 (1.5 ppm)	0 ± 0	100 ± 0 a	0 ± 0	100.0 a
*Alternaria solani* ITCC-3640	Blank NPs N 1.0 (1.0 ppm)	32.5 ± 3.5	50 ± 3.5	--	--
	Loaded NPs NF 0.5 (0.5 ppm)	21 ± 1.4	67.7 ± 1.4 bc	10.5 ± 0.7	83.8 ± 0.7 b
	Loaded NPs NF 1.0 (1.0 ppm)	21 ± 0	67.7 ± 0 bc	10 ± 0	84.6 ± 0 b
	Loaded NPs NF 1.5 (1.5 ppm)	11 ± 0	83.1 ± 0 b	10 ± 0	84.6 ± 0 b
*Sclerotonia sclerotiorum* ITCC-5492	Blank NPs N 1.0 (1.0 ppm)	13.5 ± 0.7	60.3 ± 0.7 c	--	--
	Loaded NPs NF 0.5 (0.5 ppm)	15.5 ± 0.7	54.4 ± 0.7 c	10.5 ± 0.7	69.1 ± 0.7 bc
	Loaded NPs NF 1.0 (1.0 ppm)	0 ± 0	100 ± 0 a	0 ± 0	100 ± 0 a
	Loaded NPs NF 1.5 (1.5 ppm)	0 ± 0	100 ± 0 a	0 ± 0	100 ± 0 a

Each value is the mean of a triplicate. Mean ± SD followed by the same letter in the column of treatment are not significantly different at *p* ≤ 0.05 as determined by a *t*-test.

**Table 4:** Encapsulation and loading capacity of mancozeb in CSCRG NPs.

Formulation	Encapsulation Efficiency (%)	Loading Capacity (%)
CSCRG-0.5	17.0 ± 1.20	87.3 ± 0.20
CSCRG-1.0	38.1 ± 0.56	95.5 ± 1.15
CSCRG-1.5	58.3 ± 0.83	92.9 ± 0.95

± = Standard deviation; n = 3.

**Table 5:** Percentage disease severity (%DS) and percentage disease control efficacy (%DCE) of nanofor-mulations (CSCRG-1.0) in pot house conditions.

Treatment	*A. alternata*		*S. lycopersici*		*A. solani*		*S. sclerotiorum*	
	% DS	% DCE	% DS	% DCE	% DS	% DCE	% DS	% DCE
Pure control C	6.1 ± 1.4	--	12.7 ± 3.5	--	4.5 ± 0.7	--	13.5 ± 2.1	--
Control CP	42.9 ± 3.3	--	40.9 ± 0.8	--	29.4 ± 1.6	--	27.4 ± 1.6	--
Fungicide F	20.8 ± 6.9	76.6 ± 5.8 a	20.2 ± 2.8	69.5 ± 1.8 a	6.7 ± 0.6	76 ± 1.1 a	9.9 ± 1	68 ± 6.9 a
Fungicide FP	27.6 ± 3.4	72.8 ± 3.5 a	12.9 ± 3.3	65.9 ± 1.1 a	9.9 ± 0.5	65 ± 2.2 b	10.9 ± 2.4	68.3 ± 3.4 a
Blank NPs N	24.6 ± 5.7	66.4 ± 5.5 b	12.2 ± 4.3	68.2 ± 4.2 a	7.4 ± 1.6	78.1 ± 2.2 a	10.9 ± 0.4	61.9 ± 5.7 b
Blank NPs NP	22 ± 0.7	60.6 ± 3.4	19.4 ± 8.3	56.8 ± 0.5 b	11.4 ± 1.3	66.1 ± 0.2	10 ± 0.6	61.4 ± 0.7 b
Loaded NPs NF	10.4 ± 2.7	70.3 ± 6.9 a	12.6 ± 4.6	73 ± 3.6 a	6.4 ± 0.6	79.4 ± 1.7 a	6.1 ± 0.2	77.2 ± 2.7 a
Loaded NPs NFP	17.4 ± 3	68.4 ± 9.8 a	12.8 ± 4	68.9 ± 0.9 b	9.6 ± 2.8	75.8 ± 1 a	7 ± 0.8	72.2 ± 3 a

Each value is a mean of a triplicate. Mean ± SD followed by the same letter in the column of treatment indicate that values are not significantly different at *p* ≤ 0.05, as determined by a Bonferroni post hoc *t*-test.

## Data Availability

The data presented in this study are available upon request from the corresponding author.

## Supplementary Materials

The following are available online at https://www.mdpi.com/article/10.3390/polym14010041/s1, Figure S1: *In vitro* antifungal efficacy of blank, and fungicide (1.0 mg/ml) loaded CSCRG NPs at three concentrations (0.5, 1.0 and 1.5 ppm) using mycelium inhibition method against pathogens of tomato (a) *A. alternata* (b) *S. lycopersici*; and *potato* (c) *A. solani* (d) *Sclerotinia sclerotiorum*; where: PC-pure control (Petri plates with PDA alone), C-control, F-commercial fungicide, N-blank CSCRG NPs, NF-fungicide loaded CSCRG NPs having 1.0 mg/ml of mancozeb, Figure S2: Treatment effects of CSCRG NPs on disease efficacy (pot conditions); in tomato plants (a) early blight and (b) leaf spot; in potato plants (c) early blight (d) stem rot.
