# Supplementary material for: Assessment of Antifungal Efficacy and Release Behavior of Fungicide-Loaded Chitosan-Carrageenan Nanoparticles against Phytopathogenic Fungi
Source: Polymers (Basel). 2021 Dec 23;14(1):41. doi: 10.3390/polym14010041 (PMC8747246; doi:10.3390/polym14010041)
Supplement: Supplementary file 1 [file polymers-14-00041-s001.zip › polymers-1509798-supplementary.pdf]

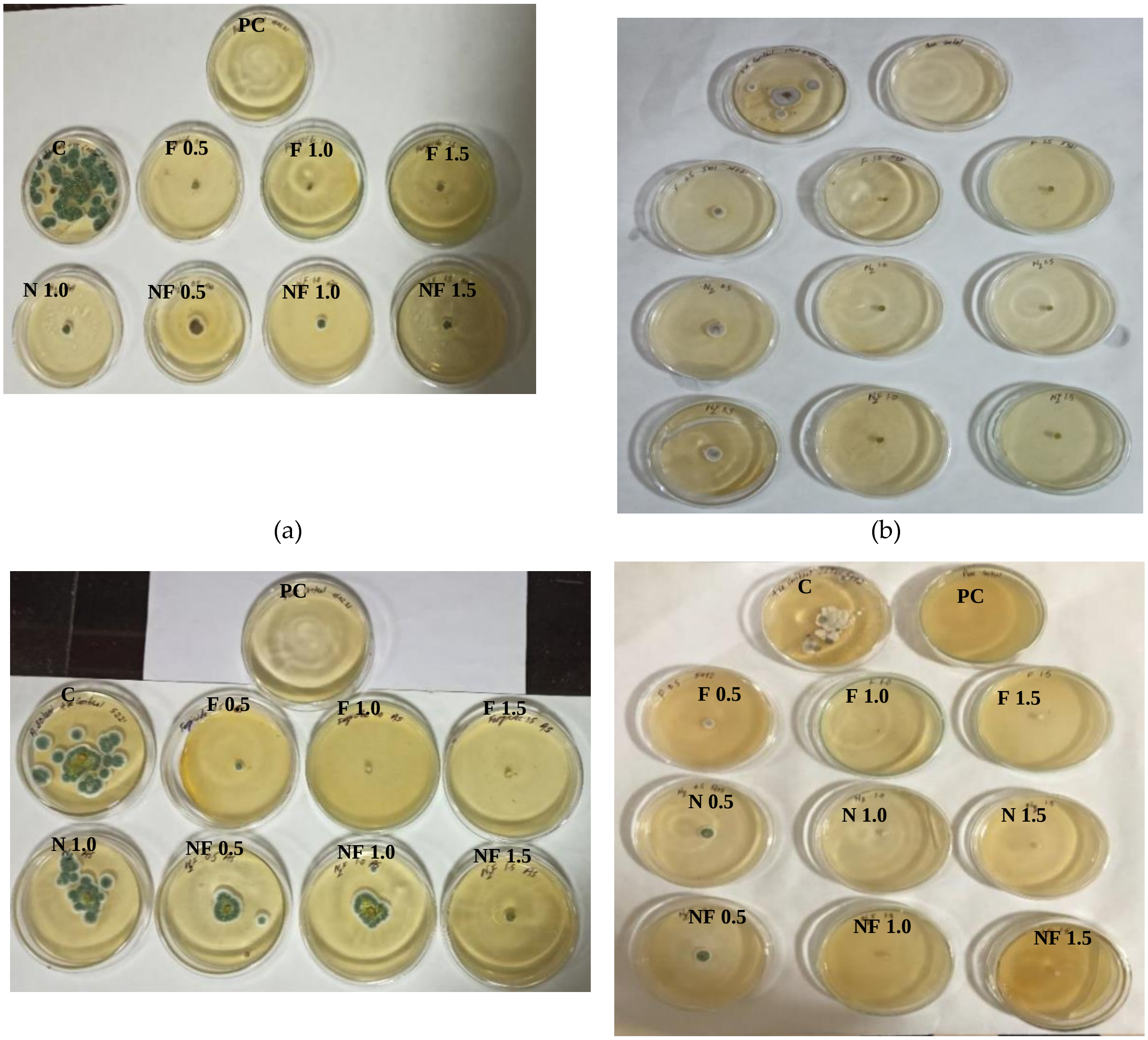

(a) (b)

(c) (d)

Figure S1. *In vitro* antifungal efficacy of blank, and fungicide (1.0 mg/ml) loaded CSCRG NPs at three concentrations (0.5, 1.0 and 1.5 ppm) using mycelium inhibition method against pathogens of tomato (a) *A. alternata* (b) *S. lycopersici; and potato (c) A. solani (d) Sclerotinia sclerotiorum*; where: PC-pure control (Petri plates with PDA alone), C-control, F-commercial fungicide, N-blank CSCRG NPs, NF-fungicide loaded CSCRG NPs having 1.0 mg/ml of mancozeb.

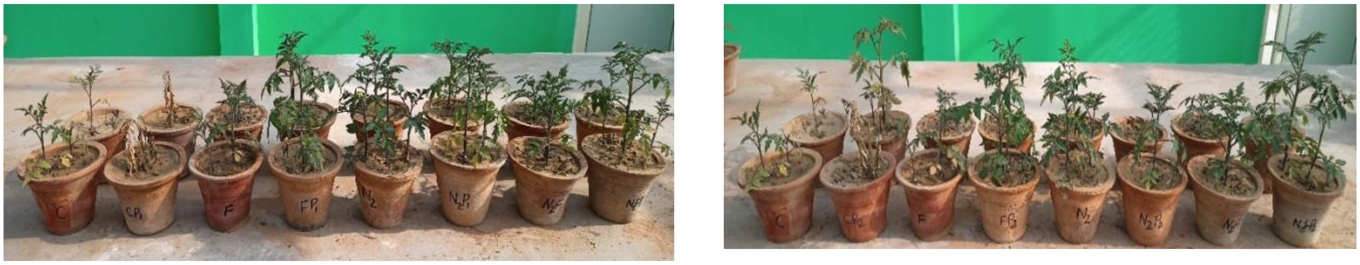

(a) **(b)**

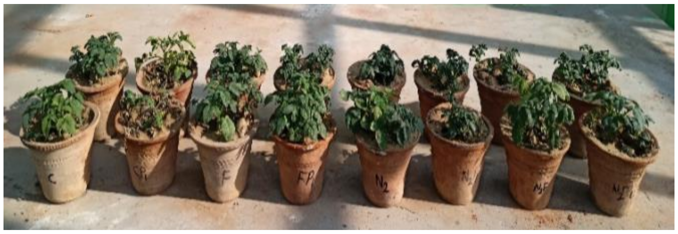

(c)

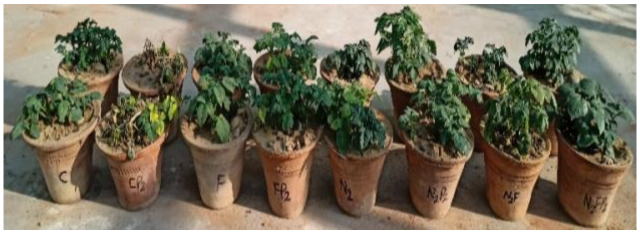

(d)

Figure S2. Treatment effects of CSCRG NPs on disease efficacy (pot conditions); in tomato plants (a) early blight and (b) leaf spot; in potato plants (c) early blight (d) stem rot.
